# Vaccine Hesitancy and Susceptibility to SARS-CoV-2 Misinformation in Japanese Youth: The Contribution of Personality Traits and National Identity

**DOI:** 10.3390/ijerph21010042

**Published:** 2023-12-27

**Authors:** Damian J. Rivers, Giancarla Unser-Schutz, Nathanael Rudolph

**Affiliations:** 1 School of Systems Information Science, Future University Hakodate, Hakodate 041-8655, Japan; 2 Department of Interpersonal and Social Psychology, Rissho University, Tokyo 141-8602, Japan; giancarlaunserschutz@ris.ac.jp; 3 Faculty of Science and Engineering, Kindai University, Osaka 577-8502, Japan; nrudolph@kindai.ac.jp

**Keywords:** Japan, misinformation, national identity, personality, SARS-CoV-2, vaccination, youth

## Abstract

During the pandemic, the Japanese government drew upon the cultural concept of *jishuku*, or personal self-constraint, requesting that individuals accept responsibility for their behaviors and consider minimizing the potential negative impact on others. While the *jishuku* approach to pandemic management rests upon the established and persuasive influence of cultural norms, variability in adherence can be expected according to age. This article documents an investigation into factors impacting vaccine hesitancy and susceptibility to SARS-CoV-2 misinformation among Japanese youth. The point of departure is the belief that attitudes and behaviors, such as those underpinning the *jishuku* approach to pandemic management, arise from within a relational framework. Therefore, developmental characteristics, such as personality traits, and in-group affinity attachments, such as facets of national identity, can be expected to function as predictors of health attitudes and behaviors. The tested structural model of hypothesized interactions accounted for 14% of the observed variance in vaccine hesitancy and 20% in susceptibility to SARS-CoV-2 misinformation. With the inclusion of gender, political ideology, and trust in government SARS-CoV-2 response as control variables, the respecified model increased the amount of variance observed in vaccine hesitancy to 30% and to 25% in susceptibility to SARS-CoV-2 misinformation. The outcomes are discussed in relation to the communication of coherent public health discourse relative to personality traits and facets of national identity.

## 1. Introduction

On 14 February 2021, the Pfizer-BioNTech vaccine was approved for administration in Japan, and this was soon followed by approval for the Takeda/Moderna vaccine on 21 May 2021 [1]. As of mid-March 2023, 82.76% (104,675,948 people) of the population has received at least one shot, with 382,415,648 doses administered nationwide. Japan has experienced a total of 72,997 SARS-CoV-2 deaths from 33,320,438 confirmed cases [2], although as of late April 2023, 87% of deaths have been among people over 70 years of age [3]. From the onset of the pandemic, young people were infected at a lower rate of transmission and exhibited fewer or less severe symptoms, leading to them being described as “asymptomatic spreaders” [4]. With a lower perception of infection and mortality risk, young people were soon condemned as less compliant with public health advisories and mass vaccination programs [5,6,7,8]. Young people received the attention of the World Health Organization (WHO) as Director Tedros Adhanom Ghebreyesus warned them to take the same precautions as other demographics, while the COVID-19 Technical Lead for the WHO Health Emergencies Programme, Maria Van Kerkhove, cautioned that young people were not immune from serious illness leading to death [9]. The attention given to young people by the WHO was also reflected within several domestic situations. In the United States, articles emphasized the attitudes and behaviors of young people who were encouraged “to realize their role in the COVID-19 pandemic” [10]. In the United Kingdom, after several months of condemnation, research initiatives started to examine the disruption caused to the daily lives of young people [11]. Now, and witnessed across multiple national contexts, research shows that young people have been subject to increases in stress, anxiety, depression, and other mental health conditions due to social distancing mandates and lockdown restrictions [12,13,14,15,16,17,18].

The pandemic has drawn attention to a milieu of social, interpersonal, and environmental variables associated with pandemic management and adherence to public health mandates. The acceptance of vaccination against communicable disease remains essential to the protection of individuals, communities, and nations. Despite the administration of 13.4 billion vaccines worldwide [19,20], SARS-CoV-2 vaccines have been met with unprecedented resistance, elevating conversations surrounding conspiracy theories and media health literacy which have unexpectedly taken center stage in public health discussions [21]. While post-industrial nations such as the United States and the United Kingdom may have infringed upon civil liberties via interventional lockdown mandates and restrictions on movement [22], Japan sought an informational approach through appeals to the cultural concept of *jishuku* or personal self-constraint. Alongside the provision of statistical information and practical health guidance, this approach requests that individual citizens accept responsibility for their behaviors and consider minimizing the potential negative impact on others [23]. While the *jishuku* approach to pandemic management rests upon the established and persuasive influence of cultural norms, variability in the acceptance of personal responsibility and behavioral modification can be expected according to age. As evidence of generational differences in attitudes and behaviors in Japan, there have been calls for policymakers to consider the varied characteristics within the younger generation when crafting targeted communication strategies to address and lessen their hesitancy toward vaccines [24].

While clinical attention prioritized the elderly as the demographic at the greatest risk of mortality, young people’s information processing abilities have been consistently tested [25] due to the widespread dissemination of misinformation through social media platforms and other channels, creating a significant public health challenge [26]. Despite being several years into the pandemic, and with mass vaccination programs being at an advanced stage of administration, discussions surrounding the antecedents of vaccine hesitancy and susceptibility to SARS-CoV-2 misinformation are expected to remain prominent within public health and environmental discourse. This article therefore documents an investigation into factors impacting vaccine hesitancy and susceptibility to SARS-CoV-2 misinformation among Japanese youth. The point of departure is the belief that attitudes and behaviors, such as those underpinning the *jishuku* approach to pandemic management, arise from within a relational framework. This relational framework represents the aspect of an individual’s self-concept that originates from their awareness of belonging to a social group, coupled with the importance and emotional meaning associated with that membership [27]. Developmental characteristics and in-group affinity attachments can therefore function as predictors of attitudes and behaviors. It is hypothesized that personality traits and facets of national identity can provide insight into the vaccine hesitancy and susceptibility to SARS-CoV-2 misinformation of Japanese youth. 

## 2. Literature Review

### 2.1. Personality Traits and National Identity

The primary developmental characteristic of personality concerns the way individuals behave, feel, and think, while personality traits represent variations in the inclination to exhibit consistent patterns of thoughts, emotions, and behaviors across individuals [28]. Individual personality traits can be understood as general tendencies that are hypothesized to influence how individuals respond to a wide variety of stimuli they come across in the environment [29]. The personality traits constituting the Five-Factor Model (FFM) [30] represent the foundation of human personality structure and provide an all-encompassing classification system for variations in individual personality traits [31]. The FFM consists of extraversion (sociality and positive affect), agreeableness (cooperation and trust), conscientiousness (orderliness and persistence), neuroticism (anxiety and depression), and intellect/openness (intellectual curiosity and creativity). While several studies using the FFM have reported significant correlations between individual traits, such correlations do not serve as evidence for the existence of a higher-order personality factor [32]. Across diverse research studies, personality has been found to predict attitudes [33,34,35,36], which are then held as reliable indicators of expected behaviors [37]. 

Personality underpins various identities, often through fluctuations in traits such as neuroticism (low) and agreeableness (high) [38]. As an individual expression of a multitude of environmental and developmental factors, national identity is informed by the personality and attitude traits that are more commonly observed within the population [39] in service of an imagined in-group. The individual components, or facets, of national identity reflect affinity attachments with the national in-group (in the case of patriotism), how the national in-group relates to the national out-group (in the case of nationalism and internationalism), or as a context-specific mediation of patriotism and nationalism (in the case of commitment to national heritage in the Japanese context). National identity has been used to better understand motives for civic involvement and as a rationale for behaviors beneficial to other in-group members [40]. Observations of the self-concept derived from an awareness of such in-group memberships and the emotional strength of those memberships have been expressed through identity theory and social identity theory [41]. The cognitive act of categorization is crucial to these theories and represents the need to view oneself and one’s in-groups in positive self-affirming terms, often in contrast to less desirable out-groups. These in-group/out-group distinctions are used to cognitively structure social interactions, attitudes, and beliefs while also providing social motives for behavior (i.e., individuals act to protect the salience of their in-group attachments). 

A particular aspect of successful in-group maintenance relative to the *jishuku* approach to pandemic management is the function of solidarity. This conceptual approach permits SARS-CoV-2 to be framed as a national threat through which governments and public officials can appeal to a shared identity to motivate citizens to think (attitude) and act (behavior) in the collective national interest. Individual thought and behavior that upholds mutual interest and advantage has been reported to protect and enhance the salience of the in-group and its dominant identities [42]. It has also been reported that public-spirited attitudes and behaviors (i.e., those that show solidarity with others) are more likely with frequent communication of what is best for the collective, a strong in-group identity, and the stigmatization of dissent or noncompliance. In the context of the pandemic, more pronounced national identities relate to support for social distancing and improved physical hygiene [43], while individuals with more pronounced national identities are also seen as more supportive of public health advisories [44] intended to protect the status of the in-group collective. These dynamics are especially applicable within the Japanese context as for centuries, a shared sense of national identity has been perpetuated through paternalistic appeals to tradition, heritage, and culture combined with a relational intergroup approach to outward comparison in service of supposed uniqueness. Within contemporary Japan, national identity remains a prominent in-group referent although variations exist relative to age [45], particularly in relation to nationalism [46] and patriotism [47,48]. These generational variations can be viewed as a threat to the salience and solidarity of the in-group as a coherent and unified national identity is critical for good governance and the facilitation of trust and cooperation among all citizens [49] in times of national crisis. 

Generational variations in in-group identity salience can also be examined through reference to personality traits which facilitate an understanding of how individuals engage in politics [50]. Personality traits provide insight into ideological beliefs and political voting preferences. Openness and agreeableness are traits commonly associated with liberal or left-wing attitudes, while conscientiousness has been consistently identified as a predictor of conservative or right-wing voting patterns [51,52,53]. Personality also provides insight into attitudes and behaviors relating to health on the premise that individuals do not perceive or respond to threat the same manner [54,55,56]. Research shows that individuals high in extraversion are more likely to smoke cigarettes, consume alcohol, and have multiple sexual partners [57]. In contrast, individuals high in conscientiousness are more likely to engage in preventative actions such as wearing seat belts, exercising, and eating a balanced diet [58]. Extraversion and conscientiousness have featured as prominent traits in a variety of studies into SARS-CoV-2 attitudes and behaviors. Individuals low in extraversion and high in conscientiousness are more likely to engage in proactive health behaviors such as handwashing and social distancing [59]. Other studies have shown that individuals low in extraversion and high in neuroticism, agreeableness, and conscientiousness are more likely to engage in positive hygiene and social distancing [60], whereas individuals low in extraversion and high in openness, agreeableness, and conscientiousness are more likely to adhere to preventative measures [61]. These studies suggest a positive role for conscientiousness and a negative role for extraversion in the maintenance of health and well-being. A recent study into the reluctance of Japanese citizens to accept vaccination and/or modify behaviors in accordance with the government request for self-restraint identified males under 30 years of age and those with higher-than-average extraversion as the most at-risk demographic [62].

### 2.2. Vaccine Hesitancy and Susceptibility to SARS-CoV-2 Misinformation

Vaccine hesitancy represents the delayed acceptance or refusal of available vaccination against communicable disease and infection [63]. The acceptance of vaccination is dependent on complex factors relating to individuals and their environment [64,65] and is inclusive of the relational dynamics and differences between generations. In a review of 15 studies relating to SARS-CoV-2 vaccine hesitancy, reasons for delay or refusal concerned questions of safety given the speed at which the vaccines were produced, questions of effectiveness given the variability in disease severity, and a general lack of trust with the official narratives offered in support of mass vaccination [66,67]. Throughout the pandemic, the Japanese media frequently noted how government officials and health experts have been frustrated by their inability to effectively communicate with the younger demographic [68]. The inability of local and national officials to communicate effectively with young people was such that vaccination incentives were given through lottery prize draws and discount shopping coupons [69]. Further reports were circulated, citing experts who blamed young people for spreading the virus while incendiary news articles condemned the social gatherings of young people [70,71,72] and bemoaned their lack of awareness as to the threat of SARS-CoV-2 [73,74]. Blame narratives casting young people as “brazen wrongdoers” and “super-spreaders” who should “shoulder a disproportionate share of the blame” for the pandemic compounded the situation [75]. Politicians and authorities corroborated in this finger-pointing, such as when Yuriko Koike, Governor of Tokyo, called out to young people to cooperate by limiting their social activities [76]. Shigeru Omi, the leading public health authority in Japan throughout the pandemic, attempted to reach out to young people by participating in a dialogue with the comedian Rintarō, where he lamented that government SARS-CoV-2 messaging was not reaching young people [77]. 

While the social activities and movements of young people were seen as reckless and inconsiderate toward other members of society, as vaccines rolled out across Japan, vaccine hesitancy became a new problem for the government to solve [78]. Public trust in vaccine safety is often cited as the most important factor in predicting vaccination uptake [79,80]. Public health scandals such as the contamination of 1.63 million recalled doses of the Takeda/Moderna vaccine, which were found to contain particles of stainless-steel, undermined trust and caused confusion and anxiety. Several awareness campaigns designed to increase public trust were launched in response on local and national government levels [81,82,83,84]. As the pandemic progressed and its impact was felt across broader sections of society, increases in psychological distress [85] and insecurity [86] among Japanese youth were reported, and after initial subsidies and government support decreased, suicide rates increased among young people and women [87]. 

While the dissemination of conspiracy theories is commonplace during times of societal crisis [88], emergent in the infancy of the pandemic and evolving over time, several conspiracy theories gained international traction. For example, many believed that SARS-CoV-2 was a hoax or a bioweapon [89], while others believed that governments were in collaboration using SARS-CoV-2 restrictions as an experiment in population control or that 5G communications networks were infecting people via airborne transmissions [90]. Homemade remedies against SARS-CoV-2 infection were also circulated online despite lacking in any scientific validation for their efficacy [91]. Alternative theories as to the origin and potential cure for the disease were also promoted by world leaders. American President, Donald Trump, repeatedly referred to SARS-CoV-2 as the “China virus” while also advocating untested and unproven treatments such as hydroxychloroquine, chloroquine, azithromycin, and remdesivir [92]. Such information is dangerous within the context of a pandemic as increased susceptibility to misinformation negatively impacts compliance with public health guidance and a willingness to accept vaccination [93]. 

Media consumption preferences have also been linked to susceptibility to misinformation [94]. A Japan-based study reported that individuals who consume information primarily through social media are prone to possessing lower levels of health literacy and a greater inclination to believe in misinformation and conspiracy theories [95]. Other studies from Japan during the pandemic have drawn attention to the role of social media in encouraging unscientific behaviors related to SARS-CoV-2, such as eating fermented soybeans as protection from infection. The authors suggest that while such behaviors are often not inherently harmful, they are often responsible for abrupt surges in panic buying leading to product shortages [96]. The Japanese media have also highlighted the importance of debunking SARS-CoV-2 misinformation online such as the belief that vaccination can lead to infertility in women, that the vaccination will make the recipient sick as it contains an active coronavirus, or that the vaccines have not been comprehensively tested due to the speed at which they were developed [97]. It is apparent that SARS-CoV-2 misinformation affects audiences and demographics differently, underscoring the need to customize communication strategies and messaging for specific populations [98].

The reviewed literature indicates that vaccine hesitancy and susceptibility to SARS-CoV-2 misinformation within youth in Japanese society are problems that threaten to undermine the *jishuku* approach to pandemic management by weakening in-group solidarity in times of crisis. SARS-CoV-2 challenges the vitality, strength, cohesion, and collective welfare of the national in-group from a multitude of perspectives. Therefore, it is anticipated that individuals with more pronounced affinity attachments can be expected to think (attitude) and act (behavior) in ways that aim to restore the vitality, strength, cohesion, and collective welfare of the national in-group though the acceptance of vaccination and the rejection of SARS-CoV-2 misinformation. Personality traits are known to inform attitudes which are then held as reliable indicators of expected behaviors, not only in relation to in-group identity preferences, but also in relation to proactive health behaviors. Within the current study, personality and national identity serve as informed reference points for the developmental and identity salience characteristics of Japanese youth relative to personal self-constraint, social responsibility, collective cooperation, and communal solidarity, all of which are crucial to the adoption of desirable SARS-CoV-2 attitudes and behaviors. 

## 3. Methods and Materials

### 3.1. Context and Participants

Approximately 1500 Japanese university students were invited to participate in the current research between 12 January 2022 and 2 January 2023. The students were enrolled at one of four Japanese universities. Potential participants were directed to an online Japanese language survey containing an explanation of the research, participation requirements and conditions, and an informed consent declaration. On commencement of the data collection, there were 24,911,367 confirmed cases of SARS-CoV-2 nationwide across Japan, a figure that had increased to 32,588,442 by the end of the data collection period. During the same period, SARS-CoV-2 deaths increased from 49,826 to 68,399 [3]. The final convenience sample comprised of 295 (58.3%) male and 205 (40.5%) female participants, while 6 (1.2%) participants identified as other. All participants were between 18 and 25 years of age (mean = 20.19/SD = 1.90). A total of 183 (36.2%) participants were enrolled at a university in western Japan, 73 (14.4%) in northern Japan, 86 (17.0%) in southern Japan, and 164 (32.4%) in eastern Japan. At the time of participation, 36 (7.1%) participants had not received a SARS-CoV-2 vaccine, 5 (1.0%) had received one vaccine dose, 137 (27.1%) had received two vaccine doses, 271 (53.6%) had received three vaccine doses, and 57 (11.3%) had received four vaccine doses. A total of 125 (24.7%) participants reported that they had been infected with SARS-CoV-2 at least once, while 381 (75.3%) participants had never been infected.

### 3.2. Data Collection Instrument

The survey instrument comprised of three primary sections containing the latent variable indicators relative to measures of personality, national identity, vaccine hesitancy, and susceptibility to SARS-CoV-2 misinformation. Directly observed variables relating to trust in the government SARS-CoV-2 response (mean = 3.47/SD = 1.19) and political ideology (mean = 3.53/SD = 0.82) were assessed on a 6-point scale, with a higher value indicating a greater degree of trust or a more right-wing political ideology. In the first section, participants were presented with the 50-item (IPIP-BFM-50) representation of the Five-Factor Personality Model [99]. The measure was inclusive of 10 indicators for each one of the five personality traits (extraversion, agreeableness, conscientiousness, emotional stability, and imagination). Items were assessed on a five-point scale ranging from “very accurate” (5) to “very inaccurate” (1) and were distributed in non-consecutive order. In the second section, participants were presented with a national identity scale which assessed the degree to which citizens affiliate themselves with the etic-emic components of Japanese national identity [41]. The current study used 18 items representing three theoretical national identity attachments, including commitment to national heritage (6 items), nationalism (6 items), and patriotism (6 items). Items were assessed on a six-point scale ranging from “strongly agree” (6) to “do not agree at all” (1) and were distributed in non-consecutive order. In the third section, participants were presented with six items drawn from a broad SARS-CoV-2 attitudinal measure [43]. The six items represented two constructs, vaccine hesitancy and susceptibility to SARS-CoV-2 misinformation, and were assessed on a six-point scale ranging from “very much applicable” (6) to “not applicable at all” (1). These items were also distributed in non-consecutive order.

### 3.3. Scale Construction and Validation

Structural equation modeling allows researchers to assess complex multivariable relationships using latent measures. In the current study, a series of confirmatory factor analysis procedures were first undertaken in IBM AMOS v.27 to validate each measurement model prior to testing the hypothesized structural model. The χ^2^ (chi-square), NC (normed chi-square), IFI (incremental fit index), CFI (comparative fit index), and RMSEA (root mean square error of approximation) at a 90% confidence interval were selected as fit criteria. While the χ^2^ represents a badness-of -fit indicator as a non-significant value is desirable [100], the χ^2^ is sensitive to large sample sizes (≥200), meaning that a significant value is not automatically indicative of a poor fitting model [101]. The NC provides a more robust measure although there exists some contention concerning appropriate cut-off values. Some authors suggest accepting values between 1 and 5 [102], while others suggest that values between 1 and 3 are indicative of an acceptable fit [103]. The IFI is a relative fit index which compares the χ^2^ for the model tested to a null model. Values ≥ 0.90 are indicative of an acceptable fit, although values closer to 0.95 are ideal. The CFI evaluates the fit of the specified solution in relation to a nested baseline model in which covariances are fixed to zero [104]. CFI values ≥ 0.90 are required to guarantee that mis-specified models are not accepted, although values ≥ 0.95 are considered ideal [105]. The RMSEA is an absolute fit index in that it assesses how far the hypothesized model is from a perfect model. Values ≤ 0.05 reflect a good fit, values between 0.05 and 0.08 reflect an adequate fit, and values between 0.08 and 0.10 reflect a mediocre fit [106].

**Figure 1:**
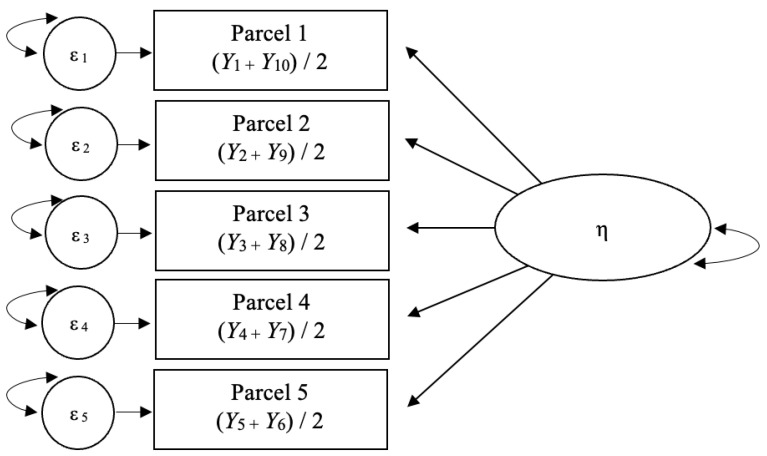
Subset-item-parcel-approach.

The original five-point scale of the IPIP-BFM-50 was first transformed to a six-point scale. The common treatment of the IPIP-BFM-50 data is to sum together the 10 indicators from each personality trait as a composite score for subsequent analysis. As the current study prioritized the modeling of latent constructs, and since an item-based approach with 10 indicators per variable is not viable [107], an alternative treatment was applied. For each personality trait, five two-item parcels were created using the mean value of items drawn from the top and bottom of each trait (see Figure 1). The practice of parceling items offers significant logistical and psychometric advantages particularly when dealing with measures containing numerous indicators. The practice is useful in stabilizing parameter estimates and improving model fit [108]. Through application of the subset-item-parcel-approach, the current study retained a focus on latent variable modelling with a permissible number of indicators while utilizing all 50-items of the IPIP-BFM-50.

The initially tested measurement model returned a poor fit to the data (χ^2^ = 1172.999 (DF = 265), *p* ≤ 0.001, NC = 4.426, IFI = 0.843, CFI = 0.842, and RMSEA = 0.082 (90% CI, 0.078, 0.087)). Examination of the standardized factor loadings revealed that several of the indicators had loadings ≤ 0.60. Two indicators were subsequently removed from each personality trait. The retested measurement model returned an acceptable goodness-of-fit (χ^2^ = 72.249 (DF = 80), *p* ≤ 0.001, NC = 3.403, IFI = 0.945, CFI = 0.944, and RMSEA = 0.069 (90% CI, 0.060, 0.078)). While the first sub-set item parcel indicator on conscientiousness (parcel 7) returned a standardized regression loading of 0.561, it was retained as conscientiousness returned sufficient average variance extracted and composite reliability values (see Table 1). Using the same subset-item-parcel-approach, three item parcels were created across each of the national identity facets. The initially tested measurement model returned an acceptable fit (χ^2^ = 71.575 (DF = 24), *p* ≤ 0.001, NC = 2.982, IFI = 0.977, CFI = 0.977, and RMSEA = 0.063 (90% CI, 0.046, 0.080)), meaning that no further re-specification was undertaken. Although the second sub-set item parcel indicator on nationalism (parcel 17) returned a standardized regression loading of 0.549, it was retained as nationalism returned sufficient average variance extracted and composite reliability values (see Table 2). Retaining a full item-based-approach with no parceling, the tested two-factor measurement model including vaccine hesitancy and susceptibility to SARS-CoV-2 misinformation returned an acceptable fit to the data (χ^2^ = 38.825 (DF = 8), *p* ≤ 0.001, NC = 4.853, IFI = 0.980, CFI = 0.980, and RMSEA = 0.087 (90% CI, 0.061, 0.116)). All indicators returned standardized loadings ≥ 0.60 and were retained.

Table 2 shows the reliability and validity attributes in addition to bivariate correlations for the variables used. As an indicator of internal consistency, all variables showed acceptable Cronbach’s alpha values between 0.73 and 0.89 [109]. Construct validity was confirmed via average variance extracted values ≥ 0.50 combined with composite reliability values ≥ 0.70 [110]. The discriminant validity of the data was confirmed through the square root of the average variance extracted values being greater than the off-diagonal correlations among the other factors. Constructs that are highly correlated (≥0.70) are suggestive of multicollinearity. Collinearity diagnostics were assessed through the variance inflation factor. All variables returned values ≤ 3.0, confirming that multicollinearity was not a serious issue in the data [111].

## 4. Results

### Structural Model Testing 

**Figure 2:**
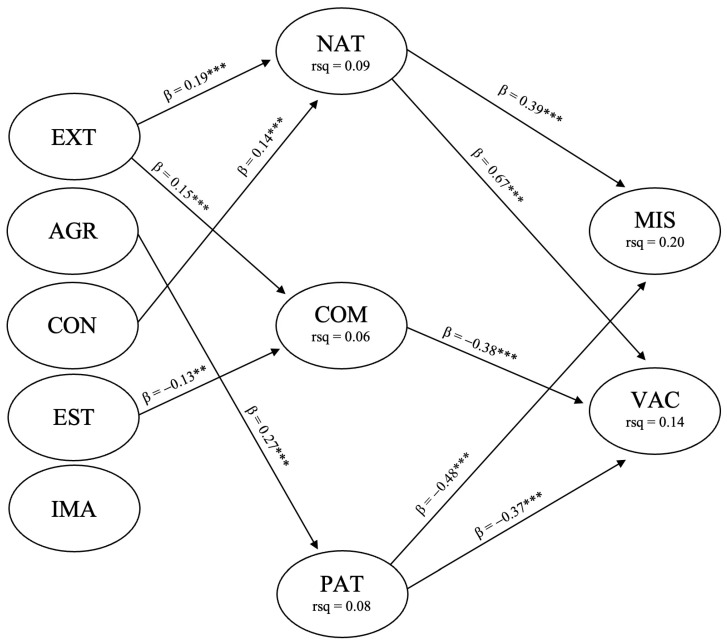
The initially tested structural model. Method ML (χ^2^ = 827.336 (DF = 370), *p* ≤ 0.001, NC = 2.236, IFI = 0.939, CFI = 0.938, and RMSEA = 0.049). EXT = extraversion, AGR = agreeableness, CON = conscientiousness, EST = emotional stability, IMA = imagination, COM = commitment to national heritage, NAT = nationalism, PAT = patriotism, VAC = vaccine hesitancy, MIS = susceptibility to SARS-CoV-2 misinformation. The permitted covariances include EXT–AGR (0.29 ***), EXT–CON (0.09 **), EXT–EST (−0.20 ***), EXT–IMA (0.35 ***), AGR–CON (0.08 ***), AGR–IMA (0.09 **), CON–EST (−0.13 ***), NAT–COM (0.46 ***), COM–PAT (0.28 ***), NAT–PAT (0.24 ***), MIS–VAC (0.23 ***). Standardized regression weights and covariances (** *p* ≤ 0.01, *** *p* ≤ 0.001).

The tested structural model consisted of ten latent variables each with three indicators. The personality trait variables (extraversion, agreeableness, conscientiousness, emotional stability, and imagination) as developmental characteristics were hypothesized to inform the facets of national identity (nationalism, commitment to national heritage, and patriotism) which, in turn, served as hypothesized predictors of the two latent attitude variables, vaccine hesitancy and susceptibility to SARS-CoV-2 misinformation. Covariances were permitted between correlated factors within but not across the boundaries of the three measurement models. The initially tested structural model returned an acceptable goodness-of-fit (χ^2^ = 827.336 (DF = 370), *p* ≤ 0.001, NC = 2.236, IFI = 0.939, CFI = 0.938, and RMSEA = 0.049 (90% CI, 0.045, 0.054)). This model is shown in Figure 2 with non-significant paths removed. 

**Figure 3:**
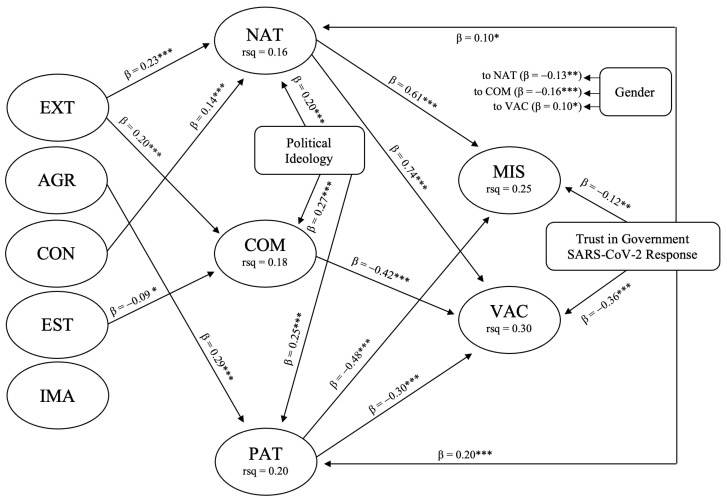
The tested structural model. Method ML (χ^2^ = 934.885 (DF = 441), *p* ≤ 0.001, NC = 2.120, IFI = 0.936, CFI = 0.935, and RMSEA = 0.047) EXT = extraversion, AGR = agreeableness, CON = conscientiousness, EST = emotional stability, IMA = imagination, COM = commitment to national heritage, NAT = nationalism, PAT = patriotism, VAC = vaccine hesitancy, MIS = susceptibility to SARS-CoV-2 misinformation. The permitted covariances include EXT–AGR (0.30 ***), EXT–CON (0.09 **), EXT–EST (−0.20 ***), EXT–IMA (0.24 ***), AGR–CON (0.08 ***), AGR–IMA (0.09 **), CON–EST (−0.13 ***), NAT–COM (0.42 ***), COM–PAT (0.21 ***), NAT–PAT (0.20 ***), MIS–VAC (0.17 ***), political ideology–government trust (0.11 **), gender–EXT (0.05 *), gender–EST (0.07 ***), gender–IMA (−0.05 *), gender–government trust (−0.06 *), gender–political ideology (−0.04 *). Standardized regression weights and covariances (* *p* ≤ 0.05, ** *p* ≤ 0.01, *** *p* ≤ 0.001).

The initially tested structural model was retested with the inclusion of three directly observed control variables (gender, political ideology, and trust in government SARS-CoV-2 response). These control variables were hypothesized to have a significant impact upon the theorized model of causal interactions. Although the respecified model returned a comparable fit to the data, the addition of the control variables contributed to a greater degree of variance being observed among the independent and dependent variables (χ^2^ = 934.885 (DF = 441), *p* ≤ 0.001, NC = 2.120, IFI = 0.936, CFI = 0.935, and RMSEA = 0.047 (90% CI, 0.043, 0.051)). This model is shown in Figure 3 with non-significant paths removed.

## 5. Discussion

The current study tested a hypothesized model of interactions intended to explain variability in vaccine hesitancy and susceptibility to SARS-CoV-2 misinformation among Japanese youth drawn from four university sites. The tested model was informed by a belief that because attitudes and behaviors arise from within a relational framework, understanding SARS-CoV-2 attitudes and behaviors among young people necessitates an exploration of developmental and identity-based variables. From the tested models, it is apparent that the five personality traits had a differentiated impact upon the three national identity facets. This provides elementary support for the idea that subscription to a specific in-group identity or affinity attachment drawn on the national level is informed by one’s own developmental characteristics. 

As demonstrated in Figure 3, the personality trait of conscientiousness shows a positive causal relationship with nationalism (β = 0.14 ***). Prior studies have cited conscientiousness as an indicator of conservative or right-wing political views and a preference for competition and order. Nationalism is a facet of national identity concerned with how the national in-group relates to the national out-group. These relational dynamics are inherently competitive as individuals seek, through processes of categorization and appraisal, to champion the position of the national in-group above, and often at the expense of, the national out-group. Nationalism is therefore a proactive and outward expression of national identity attachment as it demands an interaction with and appraisal of other nations. The foundation of this interaction and appraisal may be drawn from a range of possible criteria, including language, culture, race, politics, economy, and infrastructure. It can therefore be anticipated that individuals higher in extraversion are more likely or willing to engage in categorization through interaction and appraisal. Figure 3 shows that extraversion also exhibits a positive causal relationship with nationalism (β = 0.23 ***). Yet, in undertaking such comparative actions, individuals are engaged in risk-taking behavior as the chance exists that an unfavorable comparison may be drawn regardless of personal bias and subjectivity (i.e., there may well be stronger, more attractive, more successful nations than one’s own). Prior research suggests that extraverted individuals are more likely to also engage in risk-taking behaviors relevant to health. While a direct path between extraversion and susceptibility to SARS-CoV-2 misinformation was not tested within the model shown in Figure 3, the correlation matrices shown in Table 2 indicate a significant correlation of 0.14 **. This correlation is larger than any of the other personality traits and suggests that extraverted individuals face greater challenges in maintaining health and making informed informational decisions during times of crisis. These observations are further complicated as extraverted individuals, due to their desire for social interaction and engagement with a broad range of people and situations, are perhaps more likely to consume information on social media and have numerous social media accounts.

Figure 3 also shows that extraversion exhibits a positive causal relationship with commitment to national heritage (β = 0.20 ***). As a cultural facet of Japanese national identity commitment to national heritage services both nationalistic and patriotic attachments. Symbols of the nation such as the national anthem and national flag may be used for the nationalistic purpose of outward national identity expression and potential conflict, or for the patriotic purpose of promoting an internal sense of affection and pride without the necessity for comparison and contrast. In fact, there is evidence that all three of the national identity facets used in the current study share considerable overlap dependent on environmental variables. Conflations have been noted in prior research, such as the subtle nature of banal nationalist practices complicating the recognition of benign patriotism (*aikoku*) in the Japanese context. This difficulty arises from the political manipulation of national symbols, such as the flag and anthem, to enforce compliance from the citizens [112]. Promoting distinctiveness in culture and history within the in-group [41] through commitment to national heritage allows a positive social identity to be achieved in the absence of favorable comparisons drawn in other areas (e.g., military and/or economic power). Although beyond the scope of the current investigation, links can be drawn to the Japanese use and promotion of culture and other forms of soft-power diplomacy on the international stage. 

Banal manifestations of patriotic attachment can be seen through the positive causal relationship between the personality trait of agreeableness and patriotism (β = 0.29 ***). One might cynically understand this as an indication that banal forms of national identity only require an absence of proactive resistance and dissent (i.e., agreeableness) in that their imposition is the otherwise the default and expected position (e.g., the flying of the national flag on a public building is normative in the same way that wishing for one’s own country to be victorious in times of war or sporting competition is normative). This outcome may also serve as caution to instances where patriotic sentiment in its most banal form is used to mask more outwardly nationalistic ambitions. Japanese historical militarism fits this profile and partly explains why contemporary Japanese citizens are extremely hesitant to be ruled by interventional dictates from political figures in times of crisis. As confirmed in various studies emergent from the pandemic, the avoidance of legally mandated lockdowns reflects the postwar citizens’ aversion to the government directly encroaching on personal freedoms [113]. Yet, Japanese citizens are susceptible to the passive acceptance of top-down impositions as the personality trait of agreeableness is a positively appraised cultural feature of individual disposition that relates to the maintenance of harmony, social order, and the avoidance of interpersonal conflict. Anxiety surrounding the contentious use and ambiguous representation of national symbols and other cultural elements may also explain that outcome that emotional stability shows a negative causal relationship with commitment to national heritage (β = −0.09 *).

The direct impact of the three national identity variables on the two criterion variables highlights an interesting dynamic relating to how nationalism and patriotism may be understood as distinct despite significant overlap in terms of the sources used in the explanation or service of each respective facet. In the current study, nationalism was indicative of increased vaccine hesitancy (β = 0.74 ***) and increased susceptibility to SARS-CoV-2 misinformation (β = 0.61 ***). This further adds indirect evidence to the existence of links between extraversion, nationalism, and risk-taking behaviors. In contrast, the current study also found that patriotism was indicative of reduced vaccine hesitancy (β = −0.30 ***) and reduced susceptibility to SARS-CoV-2 misinformation (β = −0.48 ***). Moreover, commitment to national heritage was indicative of reduced vaccine hesitancy (β = −0.42 ***). These interactions and the contrasting influence of nationalistic and patriotic sentiment on the criterion variables draw attention to the ways which different forms of national identity attachments respond to in-group threats. For example, it can be argued that nationalists are likely to frame SARS-CoV-2 as an external (foreign) threat to Japanese society and thus react in an aggressive or combative way that aims to deny or undermine the legitimacy or seriousness of the threat (e.g., through increased vaccine hesitancy and increased susceptibility to SARS-CoV-2 misinformation). Similar connections between national identity, vaccine hesitancy, and misinformation have been made in a recent Polish study where it was reported that increased national narcissism (comparable with a traditional measure of nationalism) was associated with greater vaccine hesitancy and the greater endorsement of conspiracy theories [114]. 

On the other hand, the threat posed by SARS-CoV-2 from the perspective of a patriot evokes a response exclusively concerned with the protection of the home society without the aggressive or combative response or rhetoric. The current study therefore suggests that patriots are more likely than nationalists to have received vaccination and are less susceptible to SARS-CoV-2 misinformation. It might also be suggested that patriotism is a form of identity attachment that places the collective above the individual in terms of responsibility and commitment, whereas nationalism might be seen as placing individual interests and beliefs above the collective. The *jishuku* approach to pandemic management, which rests upon the established and persuasive influence of cultural norms, therefore seems dependent upon the existence of patriotic sentiment among the population as one such cultural norm. Instilling patriotism as a normative feature within Japanese citizens has a long and complex history. Some have proposed that, aside from totalitarian states, Japan has systematically employed schools for political indoctrination more than any other modern nation. The same author goes on to explain that the architects of the modern school system were mindful of issues related to morality and patriotism [115]. While such historical narratives serve a limited purpose in relation to contemporary initiatives, the participants in the current study would have all been part of the original 2006 revision of the 1947 Fundamental Law of Education that enshrined “the importance of teaching love for country and region and Japanese culture and traditions with special emphasis on moral education” [46] (p. 436). While moral education (*dōtoku*) was consequently established as a supplementary subject at elementary schools, in 2018, it was made into a formal subject [47]. 

Concerning the three control variables used in the current study (gender, political ideology, and trust in government SARS-CoV-2 response), several significant relationships were identified. Within the tested model, significant gender differences were shown in terms of nationalism (β = −0.13 **) and commitment to national heritage (β = −0.16 ***), with both identity facets being weaker among females. Unlike within many Western societies, the division of social roles and expectations based on gender remains readily apparent within Japanese society and relates to historical duties in service of the nation. In short, males have almost exclusive moral responsibility for dealing with threats to the nation (i.e., fighting in wars and protecting the economy), whereas females have the responsibility for raising children, supervising education, and ensuring the next generation is ready to serve the nation [116,117]. Given the relationship between nationalism and vaccine hesitancy, it was also expected in the current study that vaccine hesitancy would be greater among male participants than female participants (β = 0.10 *). The current study further found that subscription to a more right-wing political ideology was positively related to greater national identity attachments inclusive of nationalism (β = 0.20 ***), commitment to national heritage (β = 0.27 ***), and patriotism (β = 0.25 ***). These outcomes raise several questions as to the relationship between political affiliation and national identity in Japan. What aspects of social life within Japan inform right-wing political ideology? Is the concept of national identity an innately right-wing proposition? Do individuals with a more left-wing political leaning identify with the traditional aspects of national identity such as those used in the current study, and if not, what are the dominant components of national identity attachments from the political left?

The current study further shows the relationship between trust in government SARS-CoV-2 response and nationalism (β = 0.10 *) and patriotism (β = 0.20 ***) as affirmative. In addition, greater trust in government SARS-CoV-2 response was indicative of lower vaccine hesitancy (β = −0.36 ***) and lower susceptibility to SARS-CoV-2 misinformation (β = −0.12 **). While these relationships may be used to highlight the importance of individuals having trust in their government in times of crisis, they might also be more indicative of the dominance of agreeableness as a prioritized personality trait within the Japanese cultural sphere. Moreover, although it is possible to view the relative success of the *jishuku* approach to pandemic management as a sign of public support for government public health policies, it is just as easily—and more persuasively—explained by social pressure to conform, hence the high-levels of cooperation with publicly visible performative acts of cooperation such as mask wearing and hand-sanitizing. While such voluntary acts of cooperation are normally associated with trust in the government, this is not reflected in the Japanese situation where people’s trust has been weakened through the economic and environmental disasters over the last 30 years [118,119,120,121].

## 6. Conclusions

This article has documented an investigation into factors impacting vaccine hesitancy and susceptibility to SARS-CoV-2 misinformation among Japanese youth. The point of departure for the current investigation was the belief that attitudes and behaviors such as those underpinning the *jishuku* approach to pandemic management arise from within a relational framework, or the aspect of an individual’s self-concept that originates from their awareness of belonging to a social group, coupled with the importance and emotional meaning associated with that membership [27]. It was therefore hypothesized that personality traits and facets of national identity could provide insight into the and vaccine hesitancy and susceptibility to the SARS-CoV-2 misinformation status of Japanese youth. The current study has shown how national identities drawn relative to nationalism are likely to put individuals and communities at greater risk of SARS-CoV-2 infection through promoting increased vaccine hesitancy and susceptibility to SARS-CoV-2 misinformation. In this regard, the nationalistic response to a perceived threat, furthered in combination with extraverted personality characteristics prone to risk-taking, appear unhelpful when dealing with a public health crisis. In contrast, the patriotic response to the perceived threat is likely to be more effective in prioritizing the collective interests of the in-group in terms of health and economy. These positive effects are heightened when combined with trust in the government SARS-CoV-2 response. In other words, individuals with more pronounced patriotic identities can be seen as more supportive of public health advisories intended to protect the status of the in-group collective. To this end, and when viewed as removed from political stigmatization, the promotion of a love of country appears to be an effective method for promoting cooperation in the national fight against SARS-CoV-2 among Japanese youth. The positive impact of this approach is expected to be intensified when agreeableness serves as a dominate personality trait, as it does within the Japanese cultural landscape, and when an individual subscribes to a more right-wing political ideology. However, government discourse must seek to establish a fine-tuned balance between earning the trust and support of youth, promoting a positive love of country, and advocating for self-constraint and collective responsibility as valued members of a clearly defined national in-group. Prior engagements in the public shaming and ridicule of Japanese youth as outlined within this article should also be curtailed as such; face-threatening behavior is likely to lead to resistance, not through overt activism, aggression, and conflict such as that often seen in Western nations, but through apathy and withdrawal.

## Figures and Tables

**Table 1:** Measurement model composition and retained item loadings (n-506).

Construct	Variable	Sub-Set Item Parcel	Factor Loading	Item(s)	Mean (SD)
Personality Traits	Extraversion	1	0.829	Item 1 + Item 2	2.92 (1.46)/2.70 (1.46)
		2	0.872	Item 1 + Item 2	3.14 (1.42)/2.97 (1.45)
		3	0.826	Item 1 + Item 2	3.20 (1.52)/3.77 (1.58)
	Agreeableness	4	0.844	Item 1 + Item 2	3.90 (1.43)/4.08 (1.25)
		5	0.784	Item 1 + Item 2	4.24 (1.25)/4.32 (1.34)
		6	0.645	Item 1 + Item 2	3.83 (1.32)/4.29 (1.21)
	Conscientiousness	7	0.561	Item 1 + Item 2	3.48 (1.32)/3.75 (1.32)
		8	0.771	Item 1 + Item 2	3.45 (1.62)/4.23 (1.32)
		9	0.800	Item 1 + Item 2	3.28 (1.47)/2.88 (1.52)
	Emotional Stability	10	0.802	Item 1 + Item 2	3.97 (1.54)/4.04 (1.51)
		11	0.786	Item 1 + Item 2	4.72 (1.31)/3.78 (1.57)
		12	0.872	Item 1 + Item 2	4.54 (1.37)/4.00 (1.40)
	Imagination	13	0.972	Item 1 + Item 2	3.35 (1.34)/3.30 (1.32)
		14	0.689	Item 1 + Item 2	4.23 (1.34)/3.05 (1.40)
		15	0.742	Item 1 + Item 2	3.07 (1.32)/3.31 (1.36)
National Identity	Nationalism	16	0.866	Item 1 + Item 2	3.47 (1.31)/3.04 (1.34)
		17	0.549	Item 1 + Item 2	3.80 (1.31)/3.75 (1.02)
		18	0.702	Item 1 + Item 2	2.37 (1.01)/3.26 (1.27)
	Commitment to National Heritage	19	0.827	Item 1 + Item 2	2.38 (1.25)/3.65 (1.20)
		20	0.828	Item 1 + Item 2	2.61 (1.31)/2.76 (1.29)
		21	0.851	Item 1 + Item 2	2.20 (1.25)/2.96 (1.34)
	Patriotism	22	0.807	Item 1 + Item 2	3.45 (1.38)/4.43 (1.02)
		23	0.753	Item 1 + Item 2	4.31 (1.32)/4.33 (1.11)
		24	0.645	Item 1 + Item 2	4.75 (0.95)/4.38 (1.11)
SARS-CoV-2 Attitudes and Behaviors	Vaccine Hesitancy		0.825	Item 1	3.11 (1.41)
			0.727	Item 2	3.27 (1.27)
			0.681	Item 3	2.64 (1.32)
	Susceptibility to SARS-CoV-2 Misinformation		0.811	Item 1	1.69 (0.97)
			0.843	Item 2	1.51 (0.88)
			0.944	Item 3	1.55 (0.89)

Note: SD = standard deviation.

**Table 2:** Reliability and validity attributes and bivariate correlations (n-506).

	M (SD)	CA	AVE	CR	DV	1	2	3	4	5	6	7	8	9	10
1	9.35 (3.47)	0.87	0.70	0.88	0.83	--									
2	12.34 (2.64)	0.80	0.58	0.80	0.76	0.38 **	--								
3	10.54 (2.84)	0.74	0.51	0.75	0.71	0.15 **	0.21 **	--							
4	12.53 (3.19)	0.74	0.67	0.86	0.81	−0.19 **	0.03	−0.18 **	--						
5	10.17 (2.90)	0.83	0.65	0.84	0.80	0.34 **	0.15 **	0.06	−0.01	--					
6	9.86 (2.44)	0.73	0.51	0.75	0.71	0.17 **	0.09 *	0.17 **	−0.07	0.04	--				
7	8.29 (2.91)	0.87	0.69	0.87	0.83	0.17 **	0.08	0.10 *	−0.13 **	0.07	0.67 **	--			
8	12.84 (2.34)	0.77	0.54	0.78	0.73	0.12 **	0.25 **	0.10 *	0.03	0.08	0.51 **	0.43 **	--		
9	9.03 (3.36)	0.78	0.55	0.78	0.74	0.03	0.07	0.10 *	0.01	−0.01	0.09 *	−0.01	−0.13 **	--	
10	4.76 (2.51)	0.89	0.75	0.90	0.86	0.14 **	−0.02	0.02	−0.12 **	0.01	0.20 **	0.22 **	−0.11 **	0.40 **	--

Note: 1 = extraversion, 2 = agreeableness, 3 = conscientiousness, 4 = emotional stability, 5 = imagination, 6 = nationalism, 7 = commitment to national heritage, 8 = patriotism, 9 = vaccine hesitancy, 10 = susceptibility to SARS-CoV-2 misinformation (M = mean/SD = standard deviation/CA = Cronbach’s alpha/AVE = average variance extracted/CR = composite reliability/DV = discriminant validity) (* *p* ≤ 0.05, ** *p* ≤ 0.01).

## Data Availability

The data may be available upon reasonable request to the corresponding author.
